# Human-Centered AI in Sleep Health Management: Scoping Review of Stakeholder Perspectives and Co-Design Practices

**DOI:** 10.2196/93779

**Published:** 2026-07-31

**Authors:** Dacheng Dai, Fangfang Xie, Jiahe Cui, Siyu Wang, Junhao Cai, Fei Yao, Guimao Wang

**Affiliations:** 1 Tuina Department, Shanghai Municipal Hospital of Traditional Chinese Medicine, Shanghai University of Traditional Chinese Medicine, No. 274, Zhijiang Middle Road, Jingan District, Shanghai, 200071, China, 86 13764628380; 2 School of Acupuncture-Moxibustion and Tuina, Shanghai University of Traditional Chinese Medicine, Shanghai, China

**Keywords:** AI, sleep medicine specialty, sleep initiation and maintenance disorders, telemedicine, wearable electronic devices, review

## Abstract

**Background:**

Sleep disorders represent a significant public health burden associated with cardiovascular and neurocognitive morbidities. While AI technologies offer potential for personalized sleep medicine, clinical integration remains limited. This translational disparity is often attributed to a lack of human-centered design, specifically insufficient stakeholder engagement in the development and implementation of these technologies. Current research frequently prioritizes algorithmic performance over usability and patient trust.

**Objective:**

This scoping review systematically maps the extent and nature of human-centered AI (HCAI) research within sleep medicine across different AI modalities, evaluating how diverse stakeholders are involved in the design, validation, and implementation of AI tools, including patients, clinicians, and technologists.

**Methods:**

Following the PRISMA-ScR (Preferred Reporting Items for Systematic Reviews and Meta-Analyses extension for Scoping Reviews) guidelines, we searched 8 databases (PubMed, Web of Science, Embase, Scopus, IEEE Xplore, ACM Digital Library, APA PsycINFO, and CINAHL) for literature published up to June 18, 2026. We identified primary research describing the design, development, or evaluation of AI technologies for sleep health with explicit human-centered components. Included studies (n=34) were categorized based on AI technology type and the method of stakeholder engagement. Data were extracted and synthesized using a thematic analysis approach.

**Results:**

Based on the included studies, the analysis reveals an uneven distribution of research focus across technological domains as descriptive patterns rather than definitive trends. Research on generative AI (GenAI) is predominantly restricted to downstream expert auditing of output accuracy (comprising 7/11, 64% of GenAI studies), with a noticeable gap in upstream participatory design involving patients. Conversely, deep learning research primarily focuses on technical explainable AI methods to address algorithmic opacity for clinicians, yet lacks progression to real-world clinical implementation. Mobile health and wearable technologies (17/34, 50%) demonstrate the most balanced HCAI ecosystem, evidencing a complete translational cycle from upstream co-design to downstream clinical implementation. Furthermore, an emerging trend is observed where AI is evolving from an automated diagnostic tool into an interactive therapeutic agent, with recent studies indicating that lay users may perceive responses from large language models as more empathetic than those from physicians.

**Conclusions:**

Lacking formal quality appraisal, our findings reflect research activity patterns rather than confirmed clinical effectiveness. Nevertheless, this scoping review innovatively applies the HCAI framework to the sleep AI lifecycle. Unlike existing reviews prioritizing algorithmic performance metrics over usability, clinical workflow integration, and patient trust, this study systematically maps these essential sociotechnical factors. It contributes to the field by revealing distinct methodological disparities and the urgent need for upstream participatory design, particularly for GenAI. In the real world, establishing standardized protocols for human-AI interaction, ensuring algorithmic transparency, and addressing demographic biases are essential to foster the clinical trust required for effective AI adoption.

## Introduction

### Rationale

Sleep disorders have emerged as a significant global public health challenge, closely linked to cardiovascular diseases, metabolic dysregulation, and neurocognitive impairment [1,2]. Although polysomnography serves as the primary reference method for diagnosis, its resource-intensive and invasive nature severely limits its accessibility in both clinical and community settings. Consequently, AI has been increasingly applied to sleep medicine to enhance diagnostic efficiency and care delivery [3]. From automated sleep-staging algorithms to remote monitoring via consumer sleep technologies and generative AI (GenAI)–driven patient education, AI is poised to advance sleep health management toward a predictive, preventive, personalized, and participatory model of medicine [4].

Recent literature has extensively documented the rapid integration of AI into sleep medicine. These recent advancements encompass a wide range of applications, from automated sleep staging and the identification of novel sleep microstructures to the usage of large language models (LLMs) for clinical narratives and patient education [5-7]. Such rapid technological evolution highlights the profound potential of AI to drive precision health, augment clinical practice, and transition sleep medicine toward a predictive and personalized care model.

The integration of LLMs introduces additional complexity to human-centered design challenges. While these models offer scalability and simulate empathetic responses [8], they also present risks regarding content accuracy, unverified clinical advice, and variability in information readability [9,10]. These factors raise significant concerns regarding patient safety and necessitate a transition from technical performance validation to the establishment of rigorous, expert-supervised auditing frameworks. In this context, sleep specialists function as critical validators, ensuring the reliability of AI-generated content, particularly for vulnerable populations such as children and older adults.

Despite these technological advancements, a significant disparity exists between the development of high-performance algorithms and their successful integration into clinical practice and patients’ daily routines. This disconnect arises from specific limitations in current technological approaches. Although deep learning models can achieve accuracy comparable to human experts in controlled settings [11], their lack of algorithmic transparency restricts interpretability, thereby reducing clinician confidence in automated decision-making [12]. Simultaneously, discrepancies often arise between objective algorithmic outputs and patients’ subjective sleep perceptions [13]. Such misalignment can lead to increased anxiety regarding sleep data accuracy and may negatively impact adherence [14]. Furthermore, AI tools that do not sufficiently incorporate the specific requirements and environmental contexts of end users frequently encounter barriers to adoption and sustained use [15].

To address these sociotechnical challenges, the framework of human-centered AI (HCAI) and participatory design methodologies have become increasingly relevant [16]. Research strategies, including stakeholder interviews, co-design workshops, and explainable AI (XAI) techniques, are being used to align technological capabilities with clinical workflow requirements and patient needs. However, existing published reviews in the field predominantly emphasize the technical efficacy and algorithmic performance of these AI models. For instance, recent systematic and scoping reviews have comprehensively evaluated the diagnostic accuracy, sensitivity, and specificity of machine learning algorithms for automated sleep staging and wearable AI for sleep apnea detection [17,18]. While these technical reviews are highly valuable, they largely overlook the essential sociotechnical dimensions required for the successful integration of AI into real-world clinical workflows. Specifically, there is a lack of comprehensive synthesis regarding human-centered design principles, such as user trust, clinical usability, and the active involvement of patients and clinicians in the development process.

### Objectives

The primary objective of this scoping review is to systematically characterize the extent and nature of HCAI and co-design methodologies within sleep health management. Rather than evaluating algorithmic accuracy, this study uniquely applies the HCAI framework to sleep medicine and synthesizes evidence to address the following inquiries: (1) which stakeholders are engaged in the development process, (2) what specific participatory methods are used, and (3) what constitutes the primary user needs, concerns, and values? Ultimately, this review aims to establish an evidence-based framework for researchers and clinicians to facilitate the translation of AI technologies into clinically integrated and user-validated interventions, thereby supporting the advancement of personalized and accessible sleep medicine.

## Methods

### Protocol and Registration

This scoping review was conducted in accordance with the methodology framework outlined by Arksey and O’Malley [19] and subsequently enhanced by the JBI (Joanna Briggs Institute) [20]. The reporting of this review strictly adheres to the PRISMA-ScR (Preferred Reporting Items for Systematic Reviews and Meta-Analyses extension for Scoping Reviews) guidelines to ensure transparency and rigor in our literature search and synthesis [21].

### Eligibility Criteria

In accordance with the JBI methodology for scoping reviews [20], the selection criteria were operationalized based on the population, concept, and context framework, aiming to establish strict boundaries for human-centric evaluation.

### Inclusion Criteria

Regarding participants (population), the review encompasses human participants representing the full spectrum of stakeholders within the sleep health ecosystem. This explicitly includes nonclinical end users and patients with sleep disorders, caregivers, alongside professional stakeholders (eg, clinicians, technologists, and researchers).

Regarding the concept, this review adopts a broad HCAI framework spanning the full lifecycle from downstream clinical validation to upstream participatory design. To be considered HCAI, studies must report empirical qualitative or quantitative data regarding human interaction with, or perception of, the AI system. We stratified included studies into 3 progressive HCAI levels: (1) expert auditing, where clinicians act as proxy users to validate AI safety and guideline concordance; (2) user experience assessment, involving direct end user feedback on usability, trust, and emotional interaction; and (3) participatory co-design, characterizing early stakeholder engagement in the developmental phase of algorithms or interventions. Borderline studies such as technical validation studies that included a brief usability survey were included only if the human-factors evaluation was formally analyzed and reported as a defined outcome, rather than merely anecdotal mentions.

Regarding the context, this review considered any setting pertinent to sleep health management, including clinical sleep laboratories, inpatient wards, home-based monitoring, and online health communities.

Regarding the types of sources, this review considered all primary study designs, including quantitative, qualitative, and mixed-methods research, as well as usability and feasibility pilot studies.

As exclusion criteria, the following were not considered: (1) studies focusing exclusively on algorithmic performance metrics or hardware validation without direct stakeholder feedback or human-factor analysis; (2) secondary literature including reviews, editorials, conference abstracts, and commentaries lacking original empirical data; (3) animal or in vitro studies; and (4) studies not published in English.

### Information Sources

A comprehensive search was performed across 8 academic databases: PubMed, Web of Science, Embase, Scopus, IEEE Xplore, ACM Digital Library, APA PsycINFO, and CINAHL. The search covered publications from database inception to June 18, 2026. In addition to the database searches, we conducted rigorous backward citation searching by manually screening the reference lists of all ultimately included studies and performed forward citation searching using Google Scholar and Scopus (Elsevier) to review subsequent studies that cited these papers.

### Search

The reporting of our search strategy was conducted in accordance with the PRISMA-S (Preferred Reporting Items for Systematic Reviews and Meta-Analyses literature search extension) guidelines [22]. Specific PRISMA-S checklist items, namely the formal peer review of the search strategy and searches of clinical trial registries, were not applicable and therefore not conducted, as they were not part of our scoping review methodology. Search terms were formulated following Cochrane [23] and JBI guidelines [24], incorporating a wide array of MeSH terms, EMTREE terms, field modifiers, and synonyms related to “artificial intelligence” (eg, deep learning and neural networks), “sleep health” (eg, sleep apnea and polysomnography), and “participatory and co-design” (eg, usability and patient participation). For example, the full electronic search strategy used for PubMed was ("Artificial Intelligence"[Mesh] OR "Machine Learning"[Mesh] OR "Deep Learning"[Mesh] OR "Neural Networks, Computer"[Mesh] OR "Natural Language Processing"[Mesh] OR "Algorithms"[Mesh] OR "Artificial Intelligence"[tiab] OR "Machine Learning"[tiab] OR "Deep Learning"[tiab] OR "Neural Network*"[tiab] OR "Generative AI"[tiab] OR "Large Language Model*"[tiab] OR "Chatbot*"[tiab] OR "Virtual Assistant*"[tiab] OR "Natural Language Processing"[tiab] OR "NLP"[tiab]) AND ("Sleep"[Mesh] OR "Sleep Hygiene"[Mesh] OR "Sleep Wake Disorders"[Mesh] OR "Sleep Apnea Syndromes"[Mesh] OR "Polysomnography"[Mesh] OR "Actigraphy"[Mesh] OR "Sleep"[tiab] OR "Insomnia"[tiab] OR "Sleep health"[tiab] OR "Sleep quality"[tiab] OR "Sleep Apnea"[tiab] OR "Circadian"[tiab] OR "Snoring"[tiab] OR "Polysomnograph*"[tiab] OR "Actigraph*"[tiab]) AND ("Research Design"[Mesh] OR "Qualitative Research"[Mesh] OR "Focus Groups"[Mesh] OR "Interviews as Topic"[Mesh] OR "Patient Participation"[Mesh] OR "Ergonomics"[Mesh] OR "Participatory"[tiab] OR "Co-design"[tiab] OR "Co-creation"[tiab] OR "Collaborative design"[tiab] OR "User-centered"[tiab] OR "Human-centered"[tiab] OR "Stakeholder*"[tiab] OR "Perspective*"[tiab] OR "Attitude*"[tiab] OR "Perception*"[tiab] OR "Qualitative"[tiab] OR "User involvement"[tiab] OR "Patient involvement"[tiab] OR "User experience"[tiab] OR "Usability"[tiab]). The detailed search strings are listed in Multimedia Appendix 1.

### Selection of Sources of Evidence

Retrieved records were imported into EndNote (Clarivate) for duplicate removal. Titles and abstracts were screened independently by 2 reviewers (DD and FX) to identify potentially relevant studies. Subsequently, the full texts of selected papers were assessed against the inclusion criteria. Any discrepancies between reviewers were resolved through discussion or consultation with a third researcher (FY).

### Data Charting Process

In accordance with the JBI methodology for scoping reviews [24], a standardized data charting tool was developed iteratively by the research team using Microsoft Excel. Before formal data extraction, a calibration exercise was conducted where 2 reviewers (DD and FX) independently piloted the charting tool on a random sample of 5 included studies. The results were compared to ensure consistency in data extraction and to refine the charting fields. Following calibration, data extraction was independently performed by 2 reviewers (DD and FX) for all included literature. Any discrepancies or coding disagreements during the charting process were discussed and resolved through consensus or by consulting a third senior reviewer (FY) when necessary. As recommended by scoping review guidelines [19], we did not calculate a formal interreviewer agreement statistic (eg, Cohen κ) for the data charting phase, as the process was iterative and consensus-driven. Furthermore, when essential information was missing or unclear in the included studies, we attempted to contact the corresponding authors for clarification.

### Data Items

The charted data items encompassed 2 main categories to align with our research objectives. First, general study characteristics were extracted, including author, year of publication, country of origin, study design, and sample size. Second, HCAI-specific variables were systematically charted, which included the AI technology type, study objectives, stakeholder characteristics, and stage of development. In addition, qualitative data regarding stakeholder needs, perceived barriers to clinical workflow integration, and specific recommendations for AI design were extracted. The data charting tool was dynamically updated throughout the extraction phase to accommodate and capture any novel themes or insights that emerged from the literature.

### Critical Appraisal of Individual Sources of Evidence

Consistent with the methodological frameworks for scoping reviews established by Arksey and O’Malley [19] and the JBI Manual for Evidence Synthesis [20], a formal quality appraisal of the included sources of evidence was not performed. The primary objective of this scoping review is to provide a comprehensive overview of the existing literature and to identify the breadth of stakeholder perspectives and co-design practices within the field of HCAI for sleep health, regardless of the methodological quality of the individual studies. This approach allows for the inclusion of a wide range of evidence types, from formative user-centered design workshops to technical validation studies, facilitating a more complete mapping of the current research landscape.

### Synthesis of Results

In strict alignment with the JBI methodology for scoping reviews, we did not conduct a meta-analysis or assess the clinical effectiveness of the interventions. Instead, the extracted data were mapped and analyzed using a combination of descriptive statistics and qualitative thematic analysis [25]. Descriptive statistics (eg, frequencies and percentages) were used to summarize the general study characteristics, including publication trends, geographical distribution, target stakeholders, and AI modalities (eg, wearable analytics, deep learning diagnostics, and GenAI). The assignment of studies to specific technology and stakeholder-engagement categories was established a priori. For the qualitative data regarding human-AI interaction, an inductive and iterative thematic analysis approach was applied to extract meaningful patterns and sociotechnical barriers. The extracted textual data were iteratively coded by the review team and categorized into four thematic domains: (1) co-design practices and user needs; (2) trust, explainability, and technical enablers; (3) expert auditing and validation of GenAI; and (4) clinical implementation and real-world feasibility.

## Results

### Selection of Sources of Evidence

**Figure 1.**
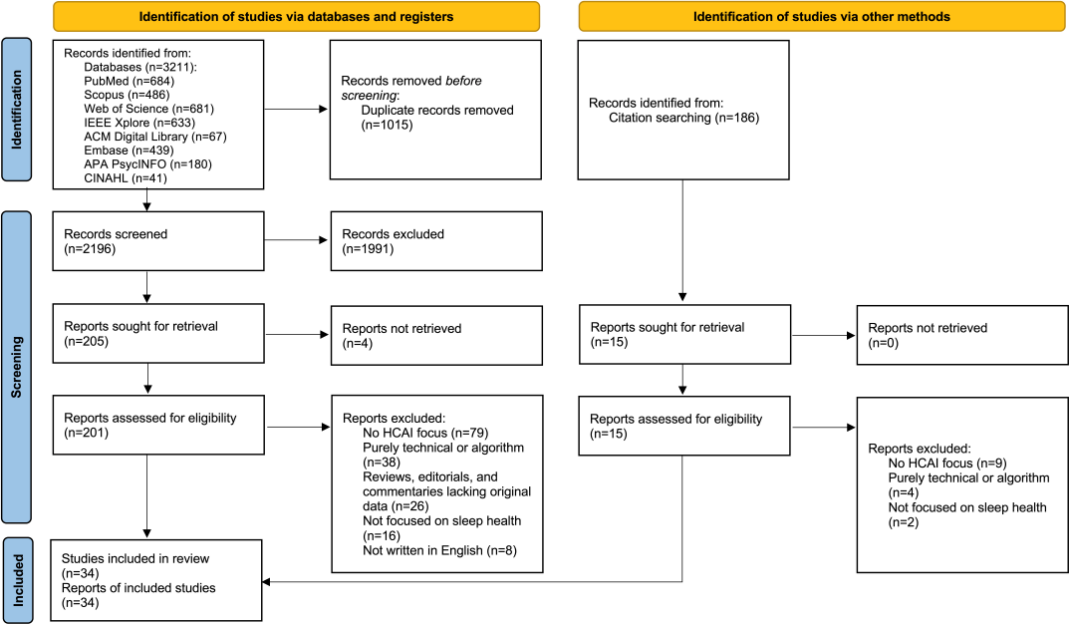
PRISMA-ScR flow diagram for study selection. This scoping review evaluates HCAI applications in sleep health management. The synthesized literature encompasses a global population of diverse stakeholders, including patients and health care professionals, with the search time frame spanning from database inception to June 18, 2026. HCAI: human-centered AI; PRISMA-ScR: Preferred Reporting Items for Systematic Reviews and Meta-Analyses extension for Scoping Reviews.

A total of 3211 records were identified through database searches. After removing 1015 duplicates, 2196 records were screened by title and abstract. We assessed 205 full-text reports against the population, concept, and context eligibility framework. Of these, 167 studies were excluded, primarily due to a lack of HCAI focus (n=79) or purely technical or algorithmic validation (n=38). Of 186 records identified through supplementary citation searching, 15 underwent full-text assessment and were all excluded (lack of HCAI focus [n=9], purely technical or algorithmic [n=4], and lacking sleep focus [n=2]), resulting in no new inclusions. Ultimately, 34 original research papers were included (Figure 1).

### Characteristics of Sources of Evidence

**Figure 2.**
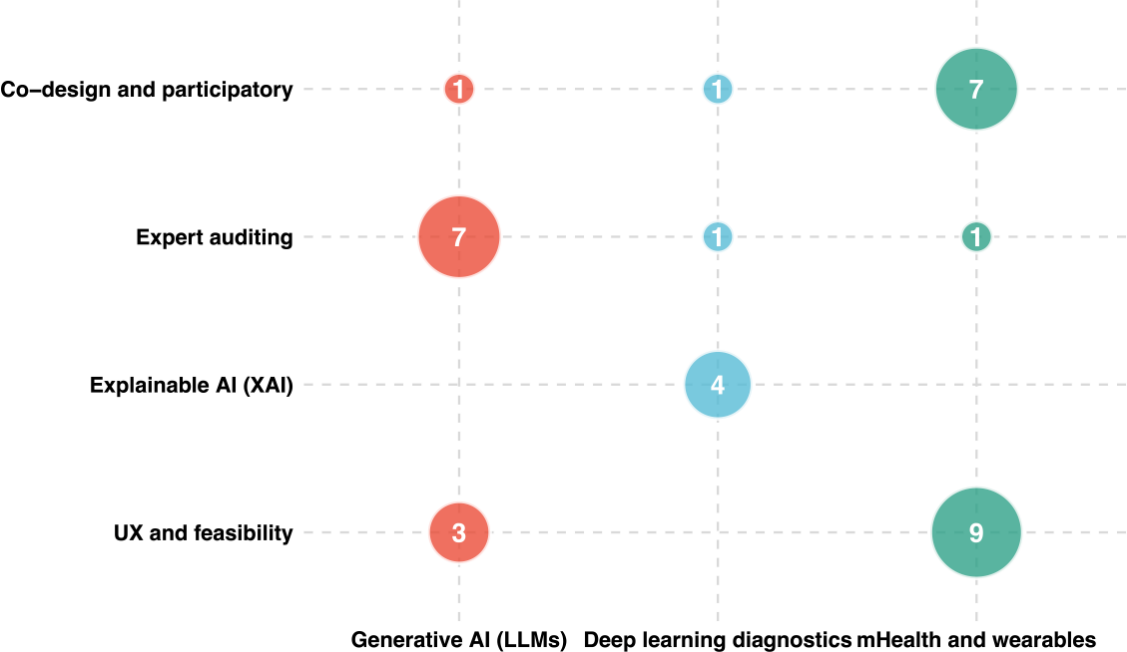
Bubble evidence gap map of HCAI research in sleep medicine. Based on a scoping review design, this figure illustrates the global distribution of evidence regarding HCAI application for sleep health management. This study’s population as mapped includes both clinical experts and patient end users, reflecting the literature published from database inception to June 18, 2026. HCAI: human-centered AI; LLM: large language model; mHealth: mobile health; UX: user experience; XAI: explainable AI.

To systematically map the research landscape, the mapped evidence is presented using a combination of descriptive narrative summaries and comprehensive data charting matrices. The general study characteristics for the included studies are outlined in Table S1 in Multimedia Appendix 2 [4,9-11,26-55], highlighting a recent publication surge in GenAI. Furthermore, to visualize the intersection of technology and human-centered design, we developed a bubble evidence gap map (Figure 2) cross-tabulating AI modalities (ie, mHealth [mobile health] and wearables, deep learning diagnostics, and GenAI) against stakeholder engagement levels. The detailed citation matrix supporting this map is provided in Table S2 in Multimedia Appendix 2.

**Figure 3.**
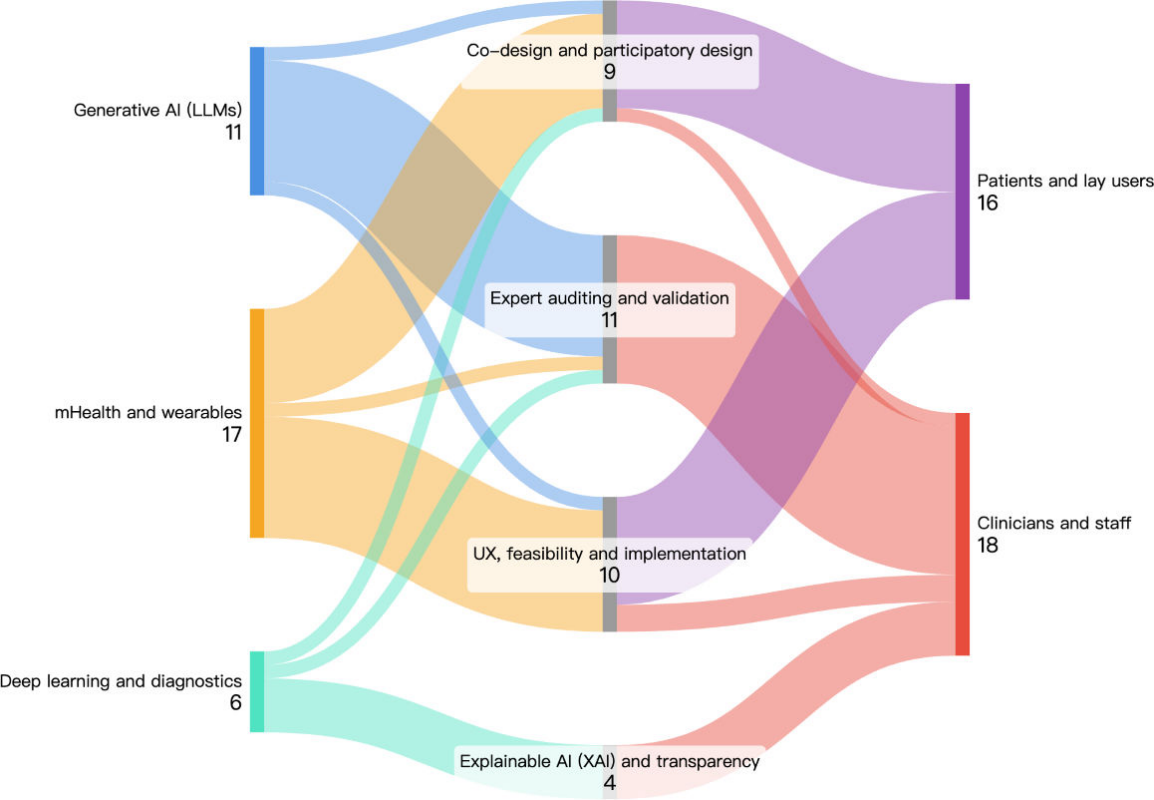
Sankey alluvial diagram mapping the multidimensional flow of HCAI research. Based on a scoping review design, this figure illustrates the structural trends of HCAI development in sleep health management. This study’s population as mapped encompasses both clinical experts and patient end users, covering the global literature published from database inception to June 18, 2026. HCAI: human-centered AI; LLM: large language model; mHealth: mobile health; UX: user experience; XAI: explainable AI.

The included studies demonstrate a significant upward trend in publication volume, with over 70% (27/34) of studies published between 2023 and 2026, reflecting the rapid integration of GenAI and advanced sensing technologies in sleep medicine. Geographically, the research output is predominantly distributed across the United States, South Korea, China, and European nations. To untangle the structural disparities between technology types and stakeholder engagement, we mapped these dimensions using a Sankey alluvial diagram (Figure 3). Framed as descriptive patterns, the included studies primarily encompassed mHealth and wearable technologies (17/34, 50%), GenAI (11/34, 32%), and deep learning diagnostic models (6/34, 18%). Notably, stakeholder engagement patterns were highly correlated with technology type. In mHealth research, 88% (15/17) of studies directly recruited patients or end users for intervention testing or co-design, including adolescent survivors of cancer. Conversely, in GenAI research, 64% (7/11) of studies relied exclusively on clinical experts to audit the accuracy of LLM outputs, lacking direct assessment from the patient perspective, although recent studies have begun addressing this gap by directly evaluating LLM-powered sleep chatbots with adult patients [26,27].

Beyond the structural mapping presented in Figure 3, a further critical appraisal of the literature reveals critical deficits in clinical and ethical readiness. Overall, 56% (19/34) of studies relied exclusively on clinical expert auditing or retrospective database validation, without recruiting actual patients with sleep disorders, including most GenAI research such as Seifen et al [9] and Howard et al [10]. This indicates that the current research landscape remains expert-centered rather than patient-centered. Furthermore, although privacy is a core HCAI principle, only 6% (2/34) of studies [28,29] explicitly reported privacy-preserving designs at the architectural level, such as localized model fine-tuning or hardware-level data filtering, with the remainder relying on standard deidentification protocols. This deficit in ecological validity and ethical design constitutes a primary structural barrier to the clinical translation of HCAI.

### Results of Individual Sources of Evidence

The specific findings, extracted themes, and targeted user needs for each of the included studies are comprehensively charted and presented in Table 1 below.

**Table 1.** Summary of key themes and qualitative findings derived from the thematic analysis. This table synthesizes the global evidence from the scoping review on sleep health management, highlighting the core thematic domains, key findings, perceived barriers, and actionable HCAI^a^ design implications for target stakeholders (patients and clinicians) from database inception to June 18, 2026.

Thematic domain	Target stakeholders	Key findings and barriers	HCAI design implications	Key studies
Co-design practices and user needs	Patients (adolescents, survivors of cancer, and adults with insomnia), lay users	Emotional resonance: users prioritize empathy and tone over raw technical accuracy, noting that overly scripted or robotic language hinders engagement. Data anxiety: raw sleep scores can induce stress (orthosomnia) if not contextualized. Literacy barriers: complex medical jargon limits engagement for younger and older users.	Persona design: develop AI personas with supportive and nonjudgmental tones to act as supportive companions. Data contextualization: provide qualitative interpretations alongside quantitative scores to reduce anxiety. Accessibility: use simplified language or visual metaphors for low-literacy groups.	Kim et al (2024) [31], Kim et al (2025) [30], Duffy et al (2025) [32], Groninger et al (2025) [28], Liang et al (2024) [33], Nagele and Hough (2024) [34], Roh et al (2026) [26], Liu and Liu (2026) [27]
Trust, explainability, and technical enablers	Clinicians (physicians and technologists)	Opacity barrier: black-box deep learning models reduce clinical confidence. Logic mismatch: algorithms often classify sleep stages differently from human heuristic rules. Uncertainty: clinicians require indicators of algorithmic confidence to trust automated scoring.	Visual evidence: implement heatmaps (eg, Grad-CAM^b^) to highlight EEG^c^ features driving the decision. Uncertainty metrics: display confidence scores to signal when human review is required. Interactive refinement: allow clinicians to modify algorithmic thresholds.	Hwang et al (2022) [35], Pei et al (2025) [11], Abdelaal et al (2025) [36], Hu et al (2025) [37], Garcia-Vicente et al (2025) [38]
Expert auditing and safety of generative AI	Clinicians	Accuracy variance: high accuracy for general advice but significant errors in complex diagnosis or treatment. Fabrication risk: tendency to generate nonexistent citations or statistics. Empathy paradox: AI is rated as more empathetic than physicians but lacks clinical safety judgment.	Human-in-the-loop: mandate expert verification for all clinical outputs. Source grounding: restrict LLMs^d^ to validated medical guidelines (retrieval-augmented generation architecture) to prevent fabrication. Prompt engineering: use standardized prompts to control reading level and tone.	Seifen et al (2025) [9], Howard et al (2024) [10], Campbell et al (2023) [39], Cheong et al (2024) [40], Bragazzi and Garbarino (2024) [41]
Clinical implementation and real-world feasibility	Health professionals (nurses and care aides)	Workflow disruption: new tools often add to the workload rather than reducing it. Surveillance concerns: continuous monitoring sensors raise privacy concerns for staff and patients. Alarm fatigue: excessive notifications lead to desensitization.	Workflow integration: automate data entry into electronic health record systems to reduce administrative burden. Privacy controls: implement physical shutters or clear on/off indicators for sensors. Actionable alerts: only notify staff for clinically significant deviations.	Acosta et al (2024) [42], Zhao et al (2026) [43], Barrera et al (2020) [44], Kubo et al (2026) [45]

a HCAI: human-centered AI.

b Grad-CAM: gradient-weighted class activation mapping.

c EEG: electroencephalogram.

d LLM: large language model.

### Synthesis of Results

#### Overview

Given the methodological and technological heterogeneity of the included studies, the descriptive and thematic analysis revealed a major imbalance in HCAI maturity across different AI modalities. The synthesized evidence is narrated below through the four overarching domains reflecting the HCAI lifecycle: (1) co-design practices and targeted user needs; (2) trust, explainability, and technical enablers; (3) expert auditing and safety of GenAI; and (4) clinical implementation and real-world feasibility.

#### Co-Design Practices and Targeted User Needs

Participatory design research reveals significant heterogeneity in functional priorities and interaction preferences across diverse stakeholder groups. End users demonstrate requirements closely tied to specific lifestyle objectives. Studies targeting adolescents [30,46] indicate a preference for gamification and metaverse elements, alongside a tendency to integrate sleep management within a broader health framework such as diet and physical activity to address obesity comorbidities. University students exhibit a unique demand to link sleep health with academic performance. Co-design workshops by Liang et al [33] highlighted suggestions for sleep-learning features such as memorizing vocabulary before bed to enhance technology acceptance. In contrast, survivors of cancer explicitly favor screen-free, voice-activated interfaces to mitigate light-induced arousal [28,47], with studies reporting adherence rates as high as 94% for voice interaction. Moreover, recent evaluations of LLM-powered chatbots introduce another distinct set of user needs among adults with insomnia and poor sleep. These users prioritize emotional resonance and conversational naturalness, valuing AI interventions that act as supportive companions with highly personalized interactions [27], while reacting negatively to language that feels overly scripted or robotic [26].

Caregivers and educators prioritize monitoring capabilities and the alleviation of parenting stress. In home and school settings, parents and school health teachers consistently agreed that effective sleep interventions must encompass comprehensive management of smartphone usage restrictions and dietary habits [30]. Research involving parents of newborns [48] found that parents of preterm infants frequently expressed physical discomfort and fatigue in human-computer interactions, indicating a requirement for more intensive emotional support compared to parents of term infants.

Professionals and shift workers prioritize efficiency and fairness. For clinicians, Abdelaal et al [36] noted that 80% of experts preferred textual summaries over raw data visualizations to optimize outpatient communication efficiency. Regarding shift-working caregivers, Kubo et al [45] identified that the core requirement for AI scheduling systems lies in the fairness of shift distribution and the physiological necessity for fatigue recovery, rather than purely operational efficiency. Furthermore, evaluations by the general public indicate that lay users perceive AI responses as superior to those of human physicians in terms of empathy, reflecting a latent demand for emotionally resonant interactions [31].

#### Expert Auditing and Validation of GenAI

Expert auditing studies indicate that GenAI demonstrates high concordance in standardized clinical tasks but significantly reduced accuracy when addressing complex cases. A proof-of-concept study conducted by Seifen and colleagues in 2025 evaluated the interpretation of polysomnographic data by ChatGPT-4o (OpenAI). This investigation revealed extremely high concordance with board-certified sleep physicians in typical obstructive sleep apnea (OSA) cases, achieving a diagnostic agreement of 97%, or 29 of 30 cases, and a 100% (30/30) agreement for treatment recommendations. However, in complex scenarios involving positive airway pressure intolerance, diagnostic concordance dropped to 70%, and therapeutic concordance fell to 44%, with the system failing to propose second-line alternatives such as hypoglossal nerve stimulation [9]. Similarly, Howard et al [10] assessed alignment with expert consensus on pediatric OSA management, while 63% of AI responses aligned closely with the expert consensus mean, defined as falling within a 1-point margin on a 9-point Likert scale; 13% deviated significantly, primarily in domains requiring nuanced clinical judgment such as surgical and medical management.

In the domain of patient education, GenAI has been verified to provide clinically accurate and highly usable information, though limitations in readability adaptation persist. Bragazzi and Garbarino [41] reported that ChatGPT-4 performed comparably to sleep experts in debunking sleep health myths, achieving excellent interrater reliability (intraclass correlation coefficient=0.83). Despite high accuracy, text complexity remains a critical barrier. Campbell et al [39] found that even when using patient-friendly prompts, the mean Flesch-Kincaid grade level of ChatGPT responses was 12.45, substantially exceeding the 6th-8th grade level recommended by the American Medical Association. Furthermore, studies focusing on insomnia queries noted that varying prompting strategies, such as comparing physician-centered against patient-centered approaches, resulted in significant fluctuations in reading grade levels, ranging from 15.4 down to 8.1, underscoring the critical role of prompt engineering in controlling output complexity [49].

Despite generally high clinical accuracy, GenAI exhibits significant issues regarding the integrity and authenticity of evidence-based referencing. Campbell et al [39] observed that ChatGPT provided references only when explicitly prompted, with citations predominantly clustering around older literature having a median publication year of 2013, reflecting a latency in training data. More critically, an audit of insomnia-related queries revealed that while 80% of references cited by ChatGPT were verified as existent, only 25% of the statistical data attributed to these sources could be corroborated by the actual texts [49]. This phenomenon of citing real papers but fabricating the statistics within them constitutes a concealed risk of misinformation, mandating rigorous source verification for AI-generated statistical data in clinical and academic applications.

#### Clinical Implementation and Real-World Feasibility

Integrating AI systems into existing clinical workflows requires rigorous alignment with the operational tempo and cognitive load of medical professionals to prevent technology abandonment. Hwang et al [35] demonstrated that interactive feature selection functions were rejected by technicians as they disrupted the rapid workflow of scoring one epoch every 5 to 10 seconds, whereas automated annotations directly superimposed on signals were successfully adopted. Abdelaal et al [36] further quantified these role-specific requirements, noting that physicians, who allocate approximately 13% of their time to direct patient interaction, necessitate highly summarized aggregated data for rapid decision-making. In contrast, nurses and health educators, who dedicate 86% of their time to patient interaction, require granular daily data to facilitate detailed patient education. In psychiatric inpatient settings, Barrera et al [44] found that optical noncontact sensors allowed nursing staff to monitor pulse and respiratory rates without disrupting patient sleep, effectively addressing the clinical issue of sleep deprivation caused by traditional manual nocturnal checks.

In real-world physical settings, the form factor of devices directly dictates their feasibility among specific populations. Implementation studies involving hospitalized geriatric patients indicate a higher acceptance of nonwearable technologies compared to wearable devices. Acosta et al [42] and Zhao et al [43] deployed AI-driven undermattress sensors in geriatric wards, finding that this contactless monitoring eliminated the physical disruption associated with nocturnal manual rounds, a feature valued by interdisciplinary staff for enhancing care quality. However, studies identified a transitional barrier regarding data trust, where clinical teams preferred to maintain traditional manual sleep logs concurrently until the accuracy of sensor data was verified [42]. In home settings, Groninger et al [28] confirmed the feasibility of a screen-free interaction environment constructed via smart speakers and smart lighting for young adult survivors of cancer. This design effectively circumvented the physiological arousal associated with screen-based light exposure.

The successful implementation of AI systems depends not only on technical performance but also on organizational adaptation and the physiological alignment of algorithmic objectives. The deployment of an AI scheduling system by Kubo et al [45] demonstrated that algorithmically minimizing the median frequency of backward rotating shifts significantly increased deep sleep duration by 5 minutes and rapid eye movement sleep by 4 minutes among caregivers. The efficacy of this intervention relied on aligning algorithmic goals with the physiological recovery needs of the workforce without increasing the burden during off-duty hours. Nevertheless, the introduction of novel technologies faces challenges related to organizational inertia; Acosta et al [42] emphasized the necessity of extensive knowledge translation strategies, including the designation of technology champions and continuous interdisciplinary training, to overcome staff resistance to automation and mitigate trust deficits caused by technical glitches.

#### Trust, Explainability, and Technical Enablers

In clinical settings, professional trust in AI systems functions as a definitive factor for technology adoption. The qualitative analysis by Abdelaal et al [36] indicates that clinician confidence is primarily contingent upon evidence of scientific validation, regulatory approval, and quantitative metrics of model accuracy. Furthermore, transparency in data acquisition is critical; professionals emphasize the necessity of verifying the user’s identity and device wear frequency to ensure that care decisions are derived from reliable patient data. Regarding GenAI, accuracy remains a central barrier to trust. While Bragazzi and Garbarino [41] noted a high concordance with an intraclass correlation coefficient of 0.83 between LLMs and experts in identifying common sleep myths, Alapati et al [49] revealed significant risks of hallucination, finding that while 80% of references cited by ChatGPT were verified as real, only 25% of the statistical data attributed to these sources could be corroborated. This uncertainty regarding factual precision restricts the independent application of GenAI in high-risk clinical decision-making scenarios.

To mitigate the lack of transparency inherent in deep learning models, several studies have developed XAI techniques designed to correlate algorithmic decision logic with physiological significance. Pei et al [11] proposed WaveSleepNet, which uses a prototype learning mechanism to match input EEG (electroencephalogram) features with learned waveform prototypes (eg, K-complexes and spindles), thereby emulating the cognitive process of sleep experts adhering to American Academy of Sleep Medicine manual scoring rules and rendering the decision basis physiologically interpretable. In sleep respiratory medicine, Garcia-Vicente et al [38] used Grad-CAM (Gradient-Weighted Class Activation Mapping) to generate heatmaps on electrocardiogram signals, localizing temporal segments that contribute most significantly to the prediction of OSA severity. Similarly, Hu et al [37] developed TSD-Net featuring a transparent scale diffusion mechanism, which enables multilevel visual traceability from individual respiratory events to overnight diagnostic conclusions; its attention mechanism accurately highlights regions with airflow reduction greater than 90% or oxygen desaturation exceeding 3%, demonstrating high concordance with clinical scoring standards. Additionally, Hwang et al [35] developed a clinical decision support system that visualizes specific waveform patterns via detection boxes and saliency highlights on EEG traces.

The efficacy of explainability is highly dependent on the user’s clinical role and experience level, necessitating alignment with the data granularity requirements of specific workflows. A controlled experiment by Hwang et al [35] demonstrated that providing visual AI explanations significantly improved sleep staging accuracy with the Macro-F1 score increase of 6.7% and interrater reliability among novice technicians with less than 5 years of experience; however, for senior technicians relying on heuristic processing, additional explanatory information was occasionally perceived as a distraction. Furthermore, Abdelaal et al [36] identified distinct requirements for data granularity across medical roles. Physicians, constrained by limited consultation time, preferred summarized textual outputs or aggregated data within 30-minute to 1-hour windows to facilitate rapid diagnostic conclusions. In contrast, health educators favored granular daily breakdowns and visual graphs to explain the causal relationship between behaviors and sleep quality to patients in detail. These disparities indicate that for effective clinical integration, technical systems must possess adaptive data presentation capabilities to align with the cognitive load requirements of diverse clinical workflows.

## Discussion

### Summary of Evidence

#### Overview

Relative to our stated objectives, this scoping review reveals significant disparities in the distribution of technology types and stakeholder engagement methods within current sleep medicine AI research. Research activities exhibit specific clustering patterns, reflecting differing priorities across technological domains.

First, in the field of GenAI, despite a marked increase in the volume of studies, methodologies are predominantly concentrated on downstream expert auditing and validation, which occupy the nascent stage of HCAI [9,10,39,41,49]. Existing literature primarily focuses on clinicians evaluating the accuracy of LLM outputs and their concordance with clinical guidelines. In contrast, participatory design research involving patients in upstream algorithm development or interaction design is scarce, with the study by Liang et al [33] being a rare exception. This indicates that current GenAI development largely follows a technology-driven path and has not yet fully integrated end user preferences and needs [56]. However, a promising methodological shift is finally emerging to address this critical gap; very recent studies [26,27] have pioneered the direct engagement of adult patients in evaluating LLM-powered chatbots, marking a crucial transition toward true user-centered validation in sleep medicine. As AI increasingly transitions from a passive analytical tool to an active conversational system for patients, ethical implications and accountability become paramount. If an AI system provides harmful behavioral advice, the liability remains a complex legal challenge. Crucially, even when a human-in-the-loop framework with rigorous expert oversight is implemented, the potential for harm persists if an AI’s erroneous advice subtly misleads the supervising clinician or if patients act on the advice before clinical verification [10]. Under current medical and legal paradigms, the ultimate accountability typically defaults to the human provider rather than the AI developer. This disproportionate shift of liability could significantly deter clinical adoption. To resolve this, future regulatory frameworks must explicitly define shared accountability models among AI developers, health care institutions, and clinical end users. Furthermore, GenAI models present a notable risk of hallucinations by occasionally fabricating nonexistent citations or medical statistics [39,41]. To address this issue without compromising patient safety, future technical developments must integrate retrieval-augmented generation (RAG) architectures [26]. Constraining the AI’s knowledge retrieval exclusively to validated clinical guidelines can effectively mitigate hallucination risks and ensure clinical safety.

Second, research on deep learning diagnostic models is highly concentrated on the technical realization of algorithmic explainability, aiming to address the lack of transparency in complex neural networks [11,35,37,38,57]. However, few studies have advanced these high-precision diagnostic models into the implementation science phase within real-world clinical settings [58]. This observation aligns directly with recent reviews emphasizing that despite the proliferation of AI in sleep medicine, the black-box nature of algorithms and the lack of routine clinical integration remain critical barriers to adoption [59]. This lack of translational research limits the application potential of advanced algorithms in everyday practice, suggesting that future developments must prioritize evaluating how clinicians practically interact with XAI outputs (eg, heatmaps) under strict time constraints.

In comparison, mHealth and wearable technologies demonstrate a more mature HCAI modality. Literature in this domain covers the complete lifecycle from early collaborative design to late-stage clinical implementation and user experience evaluation [50-52]. Notably, the study by Kubo et al [45] demonstrates the feasibility of constructing algorithmic models through participatory methods, providing a methodological reference for the development of other AI modalities. This human-centered maturity provides a striking contrast to the broader trends identified in existing literature. For instance, recent comprehensive reviews on wearable sleep AI by Abd-alrazaq et al [18] and Aziz et al [17] revealed that the vast majority of studies exclusively prioritize diagnostic accuracy and model performance using closed datasets, largely neglecting upstream patient co-design. Our findings imply that the participatory methodologies successfully pioneered in mHealth not only enhance user trust and adherence but also serve as an urgent blueprint for the future development of more complex deep learning and GenAI systems.

#### Evolution From Automated Diagnostics to Interactive Therapeutic Support

Historically, AI in sleep medicine has primarily served as an automated diagnostic aid, with the core objective of enhancing the precision and efficiency of polysomnography scoring [60]. This review confirms that this research trajectory remains active; recent works by Pei et al [11] and Hu et al [37] demonstrate that by enhancing algorithmic transparency, deep learning models can achieve scoring concordance approaching that of human experts.

However, with the introduction of GenAI technologies, the field is undergoing a paradigm shift toward interactive therapeutic support. The driving force behind this shift lies in the ability of LLMs to simulate human conversation and emotional expression. Kim et al [31] found that lay users rated ChatGPT’s responses to sleep health inquiries as having greater empathy and emotional support than those from human experts. This finding suggests that the function of AI is transcending mere data analysis and is gradually acquiring the potential to establish a therapeutic alliance in behavioral interventions.

The voice interaction system developed by Groninger et al [28] and the research on therapeutic story generation by Schlarb and Faber [53] further corroborate this trend. These technologies not only provide information but also attempt to enhance patient adherence through personalized interaction forms. Nonetheless, Schlarb and Faber [53] also noted that current AI-generated content still falls short of human therapists in terms of narrative depth and complex emotional processing. Therefore, the future developmental direction may involve constructing a hybrid care model: using AI to provide scalable basic emotional support and health education, while reserving complex clinical judgment and deep psychological intervention for human experts [61].

#### Determinants of Trust, Interpretation Discrepancies, and Safety Oversight

Trust has been identified as a critical variable for AI integration in clinical settings, with its determinants varying among stakeholders [62]. For clinicians, trust is primarily founded on algorithmic transparency. Studies by Hwang et al [35] and García-Vicente et al [38] indicate that unless algorithms can provide visualized decision logic (such as heatmaps of EEG features or probability scores), clinical professionals find it difficult to accept automated diagnostic recommendations.

For patients, the establishment of trust is often influenced by the consistency between subjective perception and objective data. Studies by Nagele and Hough [34] and Jang et al [54] highlight the phenomenon of sleep state misperception, where significant discrepancies exist between objective monitoring data and patients’ subjective feelings of fatigue. Deng et al [29] further quantified this discrepancy through sentiment analysis, finding that emotional tendencies in clinical narratives often do not align with physiological metrics (such as multiple sleep latency test results). This suggests that AI systems relying solely on objective data may lead to the neglect of patient experiences, thereby reducing user trust.

Furthermore, the widespread application of GenAI introduces new safety challenges. Although Seifen et al [9] showed high diagnostic accuracy of AI in simple cases, Alapati et al [49] and Howard et al [10] pointed out the risk of LLMs generating fictitious references and incorrect medical advice. Therefore, a rigorous expert oversight mechanism must be maintained in clinical applications. AI should be positioned as a tool for drafting or preliminary screening, with final clinical decisions validated by qualified medical professionals [63].

#### Challenges in Ecological Validity and Clinical Integration

Although AI technologies perform exceptionally well in controlled experimental environments, this review finds that their application in real-world settings still faces significant challenges [64]. Most included studies remain in the prototype development or offline validation phases, lacking field deployment studies with high ecological validity.

Implementation studies by Acosta et al [42] and Barrera et al [44] in hospital wards and psychiatric settings reveal that the introduction of technology is a complex sociotechnical process. For instance, while mattress sensors can provide continuous physiological data, they also raise concerns among nursing staff regarding alarm fatigue and patient privacy breaches. Zhao et al [43] further noted that technological intervention might alter existing health care workflows, resulting in new workload burdens. These findings underscore that technical accuracy alone is insufficient to guarantee clinical adoption. Future research must adopt implementation science frameworks to systematically evaluate the compatibility of technology with clinical workflows, organizational culture, and physical environments [65].

#### Future Research Directions

Regarding GenAI, future research agendas must transition from the exclusive post hoc validation of output accuracy to upstream participatory prompt engineering [66]. While recent studies [26,27] have pioneered this transition by directly testing LLM sleep chatbots with patients, the majority of current expert auditing studies [9,10] focus on factual concordance, often overlooking lay preferences for emotional support [31]. Future inquiries should engage patients and multidisciplinary teams early in the development cycle through iterative co-design workshops to construct and optimize prompt libraries. This ensures that the linguistic style of AI-generated advice aligns with patient emotional needs and health literacy levels while maintaining clinical precision.

The stagnation in deploying deep learning diagnostic models necessitates a shift from technical performance evaluation to clinical implementation science [67]. It is recommended to adopt a dual-layer evaluation framework to guide technology translation: at the organizational level, the Consolidated Framework for Implementation Research should be applied to systematically identify workflow compatibility and institutional barriers, as demonstrated by Acosta et al [42] in sensor deployment; at the individual level, the technology acceptance model or the unified theory of acceptance and use of technology should be integrated to quantify clinicians’ perceived usefulness and ease of use regarding AI-assisted decision-making [67]. Such structured implementation research is critical for identifying and addressing sociotechnical barriers hindering the normalization of HCAI.

Given the limitations of AI in handling complex cases, establishing standardized protocols for human-AI collaboration is urgent [68]. Future system designs should prioritize uncertainty quantification interfaces that visually communicate prediction confidence levels to clinicians [37]. Research emphasis should be placed on developing human-centered interaction mechanisms that allow experts to correct low-confidence AI suggestions and feed these corrections back into the model for continuous learning. This reciprocal model of hybrid intelligence contributes to maintaining diagnostic efficiency while ensuring the safety and accountability of final clinical decisions [68].

Finally, future HCAI designs must address the significant demographic biases and digital equity issues identified in current literature [69]. As existing studies predominantly focus on younger, technology-proficient populations [33], there is an urgent need to design age-friendly AI interventions. Given that older adults represent the primary demographic affected by sleep disorders [70], future trial designs must intentionally recruit older adults and individuals with lower digital literacy to enhance ecological validity. To minimize operational burden and ensure equitable access to sleep health services for this specific population, technological development should shift from screen-centric interfaces to 3 actionable, low-barrier interaction paradigms: (1) invisible passive sensing, prioritizing environmental devices such as undermattress sensors to eliminate the operational burdens of wearables [45,49]; (2) ambient interaction, using intuitive environmental cues such as smart lighting shifts instead of high-cognitive-load graphical interfaces [28]; and (3) natural language explanation, leveraging LLMs to translate complex polysomnographic metrics into accessible textual summaries, thereby bridging the interpretation gap for lay users [31,36].

### Limitations

This review is subject to several limitations that warrant consideration when interpreting the findings. First, consistent with the methodological standards for scoping reviews, a formal quality appraisal or risk of bias assessment of the included studies was not performed. While this approach facilitated the comprehensive inclusion of diverse study designs, ranging from technical prototyping to qualitative workshops and pilot trials, it implies that the findings presented reflect the landscape of research activity rather than the confirmed clinical efficacy of interventions. Consequently, the reliability of our thematic synthesis might be influenced by the heterogeneous quality of the underlying evidence, as several included studies were merely early-stage prototypes or small-sample pilot trials. Specific claims regarding health outcomes should be interpreted with caution until validated by rigorous randomized controlled trials.

Second, the existing evidence base exhibits significant demographic selection bias and a lack of clinical validation. A substantial proportion of studies relied exclusively on expert auditing or retrospective databases without patient enrollment. Among those that did recruit participants, the samples were heavily skewed toward younger, educated, and technology-proficient populations (Liang et al [33] and Griffith et al [55]). This limits the generalizability of findings to older adults, who represent the primary demographic for sleep disorders and often possess lower digital literacy levels [71]; thus, the barriers they face may be underestimated.

Third, there is a paucity of longitudinal data regarding long-term adherence. Most implementation studies were restricted to short-term feasibility periods. For example, studies by Groninger et al [28] and Acosta et al [42] focused on brief deployment windows. This cross-sectional nature of current research fails to capture the usage patterns after the novelty effect dissipates [72] and cannot adequately assess sustained user engagement for chronic disease management over time.

Fourth, a fundamental challenge remains regarding the reference standard used to train diagnostic algorithms. Most AI models are trained on data scored by human experts, yet interscorer variability in sleep staging is inherent. As noted by Pei et al [11], high algorithmic accuracy may in some instances reflect the replication of human consensus biases rather than the capture of absolute physiological truth.

Finally, the rapid evolution of GenAI poses a threat to the temporal validity of specific findings. Several included studies evaluated earlier iterations of LLMs. Given the fast-paced release of newer, multimodal models, findings related to the limitations of text-only models in accuracy or empathy may quickly become obsolete, necessitating continuous and dynamic re-evaluation mechanisms within the field. Furthermore, future implementations using RAG or real-time web search integrations may mitigate the current risks of fabricated references and uncorroborated statistical data identified in this review. Additionally, while the inclusion of the latest 2026 literature captures the forefront of patient-facing LLM chatbots, these interventions currently remain at the stage of short-term feasibility and usability pilot studies with relatively small cohorts. Future longitudinal, large-scale randomized controlled trials are required to establish their sustained clinical efficacy and long-term user adherence.

### Conclusions

This scoping review innovatively evaluated the entire sleep AI lifecycle through a multidimensional HCAI framework. Unlike existing reviews that focus on purely technical performance evaluation, this study differentiates itself by emphasizing the construction of sociotechnical systems for effective integration. We must emphasize that the included studies are highly heterogeneous. Therefore, limited by the absence of a formal quality appraisal, our findings reflect current patterns of research activity rather than confirmed clinical effectiveness. Based on this evidence base, we found that although GenAI shows the potential for continuous empathetic support, current research is predominantly limited to post hoc validation of output accuracy, lacking upstream patient participatory design. Encouragingly, recent pilot studies have begun to address this gap by evaluating chatbots directly with end users. Similarly, despite technical breakthroughs in explainable deep learning diagnostics, these models have rarely been translated into practical clinical tools that improve workflows. Conversely, mHealth and wearable technologies exhibit the most mature translational model, demonstrating that integrating user needs is critical for enhancing clinical utility. To successfully transition these early-stage prototypes into trusted clinical interventions, we explicitly separate our findings from the following broader recommendations for the field. First, developers must pivot from expert-centric validation to inclusive, participatory design. Second, it is essential to establish standardized interaction protocols to clarify human-AI accountability, adopt RAG architectures for safety, promote digital inclusion for older adults, and use implementation science frameworks to systematically assess the adaptability of technologies within complex health care environments. Only when founded on transparency, safety, and user trust can AI technologies truly serve the clinical practice of personalized sleep medicine.

## Supplementary material

10.2196/93779 Multimedia Appendix 1 Detailed search strategy.

10.2196/93779 Multimedia Appendix 2 Detailed findings.

10.2196/93779 Checklist 1 PRISMA-ScR checklist.

## Abbreviations

EEG: electroencephalogram
GenAI: generative AI
Grad-CAM: gradient-weighted class activation mapping
HCAI: human-centered AI
JBI: Joanna Briggs Institute
LLM: large language model
mHealth: mobile health
OSA: obstructive sleep apnea
PRISMA-S: Preferred Reporting Items for Systematic Reviews and Meta-Analyses literature search extension
PRISMA-ScR: Preferred Reporting Items for Systematic Reviews and Meta-Analyses extension for Scoping Reviews
RAG: retrieval-augmented generation
XAI: explainable AI
