# Supplementary material for: Human-Centered AI in Sleep Health Management: Scoping Review of Stakeholder Perspectives and Co-Design Practices
Source: J Med Internet Res. 2026 Jul 31;28:e93779. doi: 10.2196/93779 (PMC13426125; doi:10.2196/93779)
Supplement: Multimedia Appendix 1 — Detailed search strategy. [file jmir-v28-e93779-s001.docx]

| **Database / Platform** | **Search String** | Records Identified (n) |
| --- | --- | --- |
| **PubMed** | ("Artificial Intelligence"[Mesh] OR "Machine Learning"[Mesh] OR "Deep Learning"[Mesh] OR "Neural Networks, Computer"[Mesh] OR "Natural Language Processing"[Mesh] OR "Algorithms"[Mesh] OR "Artificial Intelligence"[tiab] OR "Machine Learning"[tiab] OR "Deep Learning"[tiab] OR "Neural Network*"[tiab] OR "Generative AI"[tiab] OR "Large Language Model*"[tiab] OR "Chatbot*"[tiab] OR "Virtual Assistant*"[tiab] OR "Natural Language Processing"[tiab] OR "NLP"[tiab]) AND ("Sleep"[Mesh] OR "Sleep Hygiene"[Mesh] OR "Sleep Wake Disorders"[Mesh] OR "Sleep Apnea Syndromes"[Mesh] OR "Polysomnography"[Mesh] OR "Actigraphy"[Mesh] OR "Sleep"[tiab] OR "Insomnia"[tiab] OR "Sleep health"[tiab] OR "Sleep quality"[tiab] OR "Sleep Apnea"[tiab] OR "Circadian"[tiab] OR "Snoring"[tiab] OR "Polysomnograph*"[tiab] OR "Actigraph*"[tiab]) AND ("Research Design"[Mesh] OR "Qualitative Research"[Mesh] OR "Focus Groups"[Mesh] OR "Interviews as Topic"[Mesh] OR "Patient Participation"[Mesh] OR "Ergonomics"[Mesh] OR "Participatory"[tiab] OR "Co-design"[tiab] OR "Co-creation"[tiab] OR "Collaborative design"[tiab] OR "User-centered"[tiab] OR "Human-centered"[tiab] OR "Stakeholder*"[tiab] OR "Perspective*"[tiab] OR "Attitude*"[tiab] OR "Perception*"[tiab] OR "Qualitative"[tiab] OR "User involvement"[tiab] OR "Patient involvement"[tiab] OR "User experience"[tiab] OR "Usability"[tiab]) | 684 |
| **Scopus** | TITLE-ABS-KEY("Artificial Intelligence" OR "Machine Learning" OR "Deep Learning" OR "Neural Network*" OR "Natural Language Processing" OR "Generative AI" OR "Large Language Model*" OR "Chatbot*" OR "Virtual Assistant*" OR "NLP") AND TITLE-ABS-KEY("Sleep" OR "Sleep Hygiene" OR "Sleep Disorder*" OR "Sleep Apnea*" OR "Polysomnograph*" OR "Actigraph*" OR "Insomnia" OR "Sleep health" OR "Sleep quality" OR "Circadian" OR "Snoring") AND TITLE-ABS-KEY("User-centered" OR "Human-centered" OR "Co-design" OR "Co-creation" OR "Collaborative design" OR "Participatory" OR "Stakeholder*" OR "User experience" OR "Usability" OR "Focus group*" OR "Qualitative" OR "Patient involvement" OR "User involvement") | 486 |
| **Web of Science** | TS=("Artificial Intelligence" OR "Machine Learning" OR "Deep Learning" OR "Neural Network*" OR "Natural Language Processing" OR "Algorithms" OR "Generative AI" OR "Large Language Model*" OR "Chatbot*" OR "Virtual Assistant*" OR "NLP") AND TS=("Sleep" OR "Sleep Hygiene" OR "Sleep Wake Disorder*" OR "Sleep Apnea*" OR "Polysomnograph*" OR "Actigraph*" OR "Insomnia" OR "Sleep health" OR "Sleep quality" OR "Circadian" OR "Snoring") AND TS=("Research Design" OR "Qualitative Research" OR "Focus Group*" OR "Interview*" OR "Patient Participation" OR "Ergonomics" OR "Participatory" OR "Co-design" OR "Co-creation" OR "Collaborative design" OR "User-centered*" OR "Human-centered*" OR "Stakeholder*" OR "Perspective*" OR "Attitude*" OR "Perception*" OR "Qualitative" OR "User involvement" OR "Patient involvement" OR "User experience" OR "Usability") | 681 |
| **IEEE Xplore** | ("Artificial Intelligence" OR "Machine Learning" OR "Deep Learning" OR "Neural Network" OR "Neural Networks" OR "Natural Language Processing" OR "Algorithms" OR "Generative AI" OR "Large Language Model" OR "Large Language Models" OR "Chatbot*" OR "Virtual Assistant*" OR "NLP") AND ("Sleep" OR "Sleep Hygiene" OR "Sleep Wake Disorder" OR "Sleep Wake Disorders" OR "Sleep Apnea" OR "Sleep Apneas" OR "Polysomnograph*" OR "Actigraph*" OR "Insomnia" OR "Sleep health" OR "Sleep quality" OR "Circadian" OR "Snoring") AND ("Research Design" OR "Qualitative Research" OR "Focus Group*" OR "Interview*" OR "Patient Participation" OR "Ergonomics" OR "Participatory" OR "Co-design" OR "Co-creation" OR "Collaborative design" OR "User-centered" OR "User-centric" OR "Human-centered" OR "Human-centric" OR "Stakeholder*" OR "Perspective*" OR "Attitude*" OR "Perception*" OR "Qualitative" OR "User involvement" OR "Patient involvement" OR "User experience" OR "Usability") | 633 |
| **ACM Digital Library** | [[Abstract: "artificial intelligence"] OR [Abstract: "machine learning"] OR [Abstract: "deep learning"] OR [Abstract: "neural network*"] OR [Abstract: "natural language processing"] OR [Abstract: "algorithms"] OR [Abstract: "generative ai"] OR [Abstract: "large language model*"] OR [Abstract: "chatbot*"] OR [Abstract: "virtual assistant*"] OR [Abstract: "nlp"]] AND [[Abstract: "sleep"] OR [Abstract: "sleep hygiene"] OR [Abstract: "sleep wake disorder*"] OR [Abstract: "sleep apnea*"] OR [Abstract: "polysomnograph*"] OR [Abstract: "actigraph*"] OR [Abstract: "insomnia"] OR [Abstract: "sleep health"] OR [Abstract: "sleep quality"] OR [Abstract: "circadian"] OR [Abstract: "snoring"]] AND [[Abstract: "research design"] OR [Abstract: "qualitative research"] OR [Abstract: "focus group*"] OR [Abstract: "interview*"] OR [Abstract: "patient participation"] OR [Abstract: "ergonomics"] OR [Abstract: "participatory"] OR [Abstract: "co-design"] OR [Abstract: "co-creation"] OR [Abstract: "collaborative design"] OR [Abstract: "user-centered*"] OR [Abstract: "human-centered*"] OR [Abstract: "stakeholder*"] OR [Abstract: "perspective*"] OR [Abstract: "attitude*"] OR [Abstract: "perception*"] OR [Abstract: "qualitative"] OR [Abstract: "user involvement"] OR [Abstract: "patient involvement"] OR [Abstract: "user experience"] OR [Abstract: "usability"]] | 67 |
| **EMBASE** | ('artificial intelligence'/exp OR 'machine learning'/exp OR 'deep learning'/exp OR 'neural network'/exp OR 'natural language processing'/exp OR 'artificial intelligence':ti,ab OR 'machine learning':ti,ab OR 'deep learning':ti,ab OR 'neural network*':ti,ab OR 'generative ai':ti,ab OR 'large language model*':ti,ab OR 'chatbot*':ti,ab OR 'virtual assistant*':ti,ab OR 'nlp':ti,ab) AND ('sleep'/exp OR 'sleep hygiene'/exp OR 'sleep disorder'/exp OR 'sleep apnea syndrome'/exp OR 'polysomnography'/exp OR 'actigraphy'/exp OR 'sleep':ti,ab OR 'insomnia':ti,ab OR 'sleep health':ti,ab OR 'sleep quality':ti,ab OR 'sleep apnea':ti,ab OR 'circadian':ti,ab OR 'snoring':ti,ab OR 'polysomnograph*':ti,ab OR 'actigraph*':ti,ab) AND ('user centered design'/exp OR 'participatory research'/exp OR 'qualitative research'/exp OR 'human-centered':ti,ab OR 'user-centered':ti,ab OR 'co-design':ti,ab OR 'co-creation':ti,ab OR 'collaborative design':ti,ab OR 'participatory':ti,ab OR 'stakeholder*':ti,ab OR 'user experience':ti,ab OR 'usability':ti,ab OR 'focus group*':ti,ab OR 'qualitative':ti,ab) | 439 |
| **APA PsycINFO** | (Abstract: "Artificial Intelligence" OR Abstract: "Machine Learning" OR Abstract: "Deep Learning" OR Abstract: "Neural Network*" OR Abstract: "Natural Language Processing" OR Abstract: "Generative AI" OR Abstract: "Large Language Model*" OR Abstract: "Chatbot*" OR Abstract: "Virtual Assistant*" OR Abstract: NLP) OR Abstract: AB (Abstract: "Artificial Intelligence" OR Abstract: "Machine Learning" OR Abstract: "Deep Learning" OR Abstract: "Neural Network*" OR Abstract: "Natural Language Processing" OR Abstract: "Generative AI" OR Abstract: "Large Language Model*" OR Abstract: "Chatbot*" OR Abstract: "Virtual Assistant*" OR Abstract: NLP) OR (Abstract: DE "Artificial Intelligence") OR (Abstract: DE "Machine Learning") OR (Abstract: DE "Neural Networks") OR (Abstract: DE "Natural Language Processing") AND (Abstract: Sleep OR Abstract: "Sleep Hygiene" OR Abstract: "Sleep Wake Disorder*" OR Abstract: "Sleep Apnea*" OR Abstract: Polysomnograph* OR Abstract: Actigraph* OR Abstract: Insomnia OR Abstract: "Sleep health" OR Abstract: "Sleep quality" OR Abstract: Circadian OR Abstract: Snoring) OR Abstract: AB (Abstract: Sleep OR Abstract: "Sleep Hygiene" OR Abstract: "Sleep Wake Disorder*" OR Abstract: "Sleep Apnea*" OR Abstract: Polysomnograph* OR Abstract: Actigraph* OR Abstract: Insomnia OR Abstract: "Sleep health" OR Abstract: "Sleep quality" OR Abstract: Circadian OR Abstract: Snoring) OR (Abstract: DE "Sleep") OR (Abstract: DE "Sleep Hygiene") OR (Abstract: DE "Sleep Wake Disorders") OR (Abstract: DE "Sleep Apnea") OR (Abstract: DE "Insomnia") AND (Abstract: "User-centered" OR Abstract: "Human-centered" OR Abstract: "Participatory" OR Abstract: "Co-design" OR Abstract: "Co-creation" OR Abstract: "Collaborative design" OR Abstract: Stakeholder* OR Abstract: "User experience" OR Abstract: Usability OR Abstract: "Focus group*" OR Abstract: Qualitative OR Abstract: "Patient involvement" OR Abstract: "User involvement") OR Abstract: AB (Abstract: "User-centered" OR Abstract: "Human-centered" OR Abstract: "Participatory" OR Abstract: "Co-design" OR Abstract: "Co-creation" OR Abstract: "Collaborative design" OR Abstract: Stakeholder* OR Abstract: "User experience" OR Abstract: Usability OR Abstract: "Focus group*" OR Abstract: Qualitative OR Abstract: "Patient involvement" OR Abstract: "User involvement") OR (Abstract: DE "Participatory Design") OR (Abstract: DE "Qualitative Research") OR (Abstract: DE "Human Computer Interaction") OR (Abstract: DE "User Centered Design") | 180 |
| **CINAHL** | XB (("Artificial Intelligence" OR "Machine Learning" OR "Deep Learning" OR "Neural Network*" OR "Natural Language Processing" OR "Generative AI" OR "Large Language Model*" OR "Chatbot*" OR "Virtual Assistant*" OR NLP OR MH "Artificial Intelligence+" OR MH "Machine Learning+")) AND XB ((Sleep OR "Sleep Hygiene" OR "Sleep Wake Disorder*" OR "Sleep Apnea*" OR Polysomnograph* OR Actigraph* OR Insomnia OR "Sleep health" OR "Sleep quality" OR Circadian OR Snoring OR MH "Sleep+" OR MH "Sleep Disorders+")) AND XB (("User-centered" OR "Human-centered" OR "Participatory" OR "Co-design" OR "Co-creation" OR "Collaborative design" OR Stakeholder* OR "User experience" OR Usability OR "Focus group*" OR Qualitative OR "Patient involvement" OR "User involvement" OR MH "Consumer Participation+" OR MH "Qualitative Studies+")) | 41 |
