# Supplementary material for: Human-Centered AI in Sleep Health Management: Scoping Review of Stakeholder Perspectives and Co-Design Practices
Source: J Med Internet Res. 2026 Jul 31;28:e93779. doi: 10.2196/93779 (PMC13426125; doi:10.2196/93779)
Supplement: Multimedia Appendix 2 — Detailed findings. [file jmir-v28-e93779-s002.docx]

**Table S1.** Summary of the included studies.

**Table S2.** Matrix of HCAI research activity by technology type and engagement method.

**Table S1.** Summary of the characteristics of all included studies. This table outlines the foundational data extracted for this scoping review on sleep health management, detailing the geographical locations (country), primary aims of the study, specific study designs, types of AI technology, target populations (sample and diverse stakeholders), and stages of development across the global literature published from database inception to June 18, 2026.

| Authors (Year) | Country | Type of AI / Technology | Aim of Study | Study Design | Sample / Stakeholders | Stage of Development |
| --- | --- | --- | --- | --- | --- | --- |
| Abdelaal et al. (2025) [36] | Qatar | Augmented AI / Wearable Analytics | Explore medical professionals' trust and needs regarding AI-generated scores and data granularity | Qualitative (Scenario-based interviews) | 23 medical professionals (physicians, educators, dietitians) | Prototype / Concept Design |
| Acosta et al. (2024) [42] | Canada | AI-driven Bed Sensor (Sleepsense) | Explore interdisciplinary team perspectives on barriers and facilitators to implementing AI sensors | Qualitative (Focus groups using CFIR framework) | 27 interdisciplinary staff (physicians, nurses, care aides) | Implementation / Clinical Integration |
| Alapati et al. (2024) [49] | USA | Generative AI (ChatGPT) | Evaluate accuracy of ChatGPT responses to insomnia queries across different prompting styles | Cross-sectional evaluation | 5 sleep medicine specialists (physicians and fellows) | Validation / Expert Auditing |
| Amorim et al. (2024) [50] | Portugal | Bayesian Network (Mobile App) | Assess the usability and predictive performance of an OSA risk assessment app in a clinical setting | Prospective usability study | 216 patients at a sleep clinic | Feasibility / Usability Testing |
| Arem et al. (2020) [47] | USA | Voice-activated Assistant (Alexa) | Explore feasibility of voice assistants for delivering Cognitive Behavioral Therapy for Insomnia (CBT-I) to cancer survivors | Mixed methods (Focus groups and prototype testing) | 25 breast cancer survivors | Prototype Development & Feasibility |
| Arnardottir et al. (2022) [4] | Europe | Machine Learning Platform (Sleep Revolution) | Describe the concept and objectives of a participatory P4 medicine approach to sleep apnea | Concept paper / Project protocol | 39 consortium partners (academic, clinical, industrial) | Concept / Strategy |
| Barrera et al. (2020) [44] | UK | AI-assisted Patient Monitoring (Oxehealth) | Explore staff experiences with vision-based patient monitoring in psychiatric wards | Qualitative interviews | Nursing staff and technicians | Implementation / Pilot |
| Bragazzi & Garbarino (2024) [41] | Italy | Generative AI (ChatGPT-4) | Evaluate AI accuracy in debunking sleep health myths compared to expert consensus | Comparative study | 10 sleep experts | Validation / Expert Oversight |
| Campbell et al. (2023) [39] | USA | Generative AI (ChatGPT) | Assess quality and readability of AI responses for OSA patient education under different prompts | Cross-sectional evaluation | 4 reviewers (residents and sleep surgeons) | Validation / Expert Auditing |
| Cheong et al. (2024) [40] | UK | Generative AI (ChatGPT vs Bard) | Compare performance of two chatbots in generating patient education material for OSA | Comparative evaluation | 2 Ear, Nose, and Throat (ENT) consultants | Validation / Expert Auditing |
| Deng et al. (2025) [29] | Switzerland | NLP (BERT-BiLSTM) | Analyze alignment between patient subjective narratives and objective sleep data | Retrospective modeling study | 100 patient records (clinical notes and PSG/MSLT data) | Model Development / Analysis |
| Duffy et al. (2025) [32] | UK / Canada | Mobile App (Sleep Solved) | Co-design educational sleep app content and features with young people | Virtual co-design (Patient and Public Involvement and Engagement) | 14 young people (16-24 years) | Prototype Design |
| García-Vicente et al. (2025) [38] | Spain / USA | Deep Learning (SleepECG-Net) | Develop an interpretable DL model for pediatric OSA using Grad-CAM for visualization | Model development and validation | Pediatric datasets | Model Development (Technical Enabler) |
| Griffith et al. (2024) [55] | USA | Digital Intervention & NLP | Evaluate user experience of a sleep intervention for heavy drinking young adults using NLP | Mixed methods (Randomized Controlled Trial exit interviews) | 118 young adults | Post-Intervention Evaluation |
| Groninger et al. (2025) [28] | USA | Voice Assistant (More SHEEP) | Develop and test a voice-activated CBT-I assistant for young adult cancer survivors | Mixed methods (Focus groups and single-arm test) | 41 cancer survivors (26 in focus groups, 15 in testing) | Prototype Development & Testing |
| Howard et al. (2024) [10] | USA | Generative AI (ChatGPT) | Evaluate alignment of AI responses with expert consensus statements on pediatric OSA | Comparative evaluation | Expert consensus statements (comparison standard) | Validation / Expert Auditing |
| Hu et al. (2025) [37] | China | Deep Learning (AIX System) | Develop a transparent human-in-the-loop AI system for sleep apnea diagnosis | Multi-center validation study | 15,807 PSG records (clinicians involved in interactive review) | Model Development / Implementation |
| Hwang et al. (2022) [35] | South Korea | XAI /Clinical Decision Support System | Develop a clinical decision support system with explainable features for sleep technicians | User-centered design and evaluation | 10 polysomnographic technicians | Prototype Evaluation |
| Jang et al. (2023) [54] | South Korea | Chatbot (Telegram) | Compare subjective sleep logs collected via chatbot versus objective Fitbit data | Comparative validation study | 543 community residents | Data Collection / Validation |
| Karlgren et al. (2022) [51] | Sweden | Sleep Tracking / Bio-hacking | Analyze how users collaborate online to modify and interpret sleep tracking technologies | Netnography | 4 online communities (Reddit) | Usage / Modification (User-driven) |
| Kim et al. (2024) [31] | South Korea | Generative AI (ChatGPT-4) | Compare expert and layperson ratings of AI versus specialist responses to sleep queries | Comparative survey | 10 evaluators (5 sleep experts, 5 laypeople) | Validation / User Perception |
| Kim et al. (2025) [30] | South Korea | Chatbot & Metaverse (MUZZIM) | Develop a smart health service for adolescents using user-centered design | User-centered design (Surveys and usability tests) | 96 participants (36 students, 30 parents, 30 teachers) | Concept / Prototype Design |
| Kubo et al. (2026) [45] | Japan | AI-driven Shift Scheduling | Assess impact of participatory AI scheduling on caregiver sleep and fatigue | Intervention study | 35 geriatric caregivers | Implementation / Intervention |
| Liang et al. (2024) [33] | Japan | GenAI / App Concepts | Explore design opportunities for sleep technology for university students | Co-design workshops | 51 university students | Concept / Ideation |
| Nagele & Hough (2024) [34] | UK | Wearable Tracker (Oura) | Critically analyze the disconnect between sleep data and subjective feeling | Autoethnography | 1 researcher (first-person perspective) | Post-Implementation Evaluation |
| Oh et al. (2022) [52] | USA | Consumer Sleep Technologies | Evaluate user perceptions and usability of various commercial sleep devices | Qualitative (Focus groups and usability testing) | 29 adults with self-reported poor sleep | Commercial Product Evaluation |
| Pei et al. (2025) [11] | China | Deep Learning (WaveSleepNet) | Develop an interpretable network that mimics expert reasoning for sleep staging | Model development | Public sleep datasets (SleepEDF, Sleep Heart Health Study) | Model Development (Technical Enabler) |
| Sangameswaran et al. (2023) [46] | USA | Mind-body Technologies | Co-design sleep and mental health technologies with adolescents | Co-design workshops | 13 adolescents (14-17 years) | Concept / Ideation |
| Schlarb & Faber (2024) [53] | Germany | Generative AI (ChatGPT) | Investigate if therapists can distinguish AI-generated from human-written therapeutic stories | Blinded comparative rating | 4 CBT therapists | Content Generation / Validation |
| Seifen et al. (2025) [9] | Germany | Generative AI (ChatGPT-4o) | Assess AI capability to interpret PSG results and suggest treatment compared to physicians | Diagnostic accuracy study | Fictitious patient cases evaluated by AI and Sleep Physicians | Validation / Expert Auditing |
| Wong et al. (2021) [48] | Singapore | Chatbot | Evaluate feasibility of a chatbot for parental stress, sleep, and feeding | Usability and feasibility study | 45 parents of preterm and term infants | Feasibility Testing |
| Zhao et al. (2026) [43] | Canada | AI-driven Bed Sensor (Sleepsense) | Explore patient and staff experiences with AI monitoring in subacute care | Qualitative descriptive | 55 participants (22 patients, 33 interdisciplinary staff) | Implementation / Feasibility |
| Roh et al. (2026) [26] | South Korea | Generative AI Chatbot (SleepPathfinder) | Examine the usability and feasibility of a CBT-I support chatbot integrating Socratic questioning and a self-decision mechanism | Usability and feasibility study | 45 participants | Prototype Evaluation / Feasibility |
| Liu & Liu (2026) [27] | USA | Generative AI Chatbot (AI Therapist) | Examine the feasibility, usability acceptability and preliminary efficacy of an AI chatbot for sleep promotion | Quasi-experimental study | 107 adults with self-reported poor sleep | Prototype Evaluation / Feasibility |

**Table S2.** Matrix of HCAI research activity by technology type and engagement method. This matrix presents the findings of our scoping review on sleep health management, mapping how globally distributed study populations are engaged across three main AI modalities within the literature published from database inception to June 18, 2026.

| Interaction Modality | Generative AI (LLMs) | Deep Learning & Diagnostics | mHealth & Wearables |
| --- | --- | --- | --- |
| Co-design & Participatory Design | Liang et al. (2024) [33] | Arnardottir et al. (2022) [4] | Kim et al. (2025) [30], Sangameswaran et al. (2023) [46], Duffy et al. (2025) [32], Groninger et al. (2025) [28], Arem et al. (2020) [47], Kubo et al. (2026) [45], Abdelaal et al. (2025) [36] |
| Expert Auditing & Validation | Seifen et al. (2025) [9], Bragazzi & Garbarino (2024) [41], Howard et al. (2024) [10], Cheong et al. (2024) [40], Alapati et al. (2024) [49], Campbell et al. (2023) [39], Schlarb & Faber (2024) [53] | Deng et al. (2025) [29] | Amorim et al. (2024) [50] |
| Explainable AI (XAI) & Transparency | N/A | Hu et al. (2025) [37], Pei et al. (2025) [11], Hwang et al. (2022) [35], García-Vicente et al. (2025) [38] | N/A |
| UX, Feasibility & Implementation | Kim et al. (2024) [31], Roh et al. (2026) [26], Liu & Liu (2026) [27] | N/A | Acosta et al. (2024) [42], Zhao et al. (2026) [43], Barrera et al. (2020) [44], Oh et al. (2022) [52], Jang et al. (2023) [54], Karlgren et al. (2022) [51], Nagele & Hough (2024) [34], Griffith et al. (2024) [55], Wong et al. (2021) [48] |
